# Supplementary material for: Investigating the population structure of Moraxella catarrhalis using a cgMLST scheme and LIN code system
Source: Nat Commun. 2025 Oct 17;16:9137. doi: 10.1038/s41467-025-64487-8 (PMC12534458; doi:10.1038/s41467-025-64487-8)
Supplement: Supplementary file 1 — Supplementary Information [file 41467_2025_64487_MOESM1_ESM.pdf]

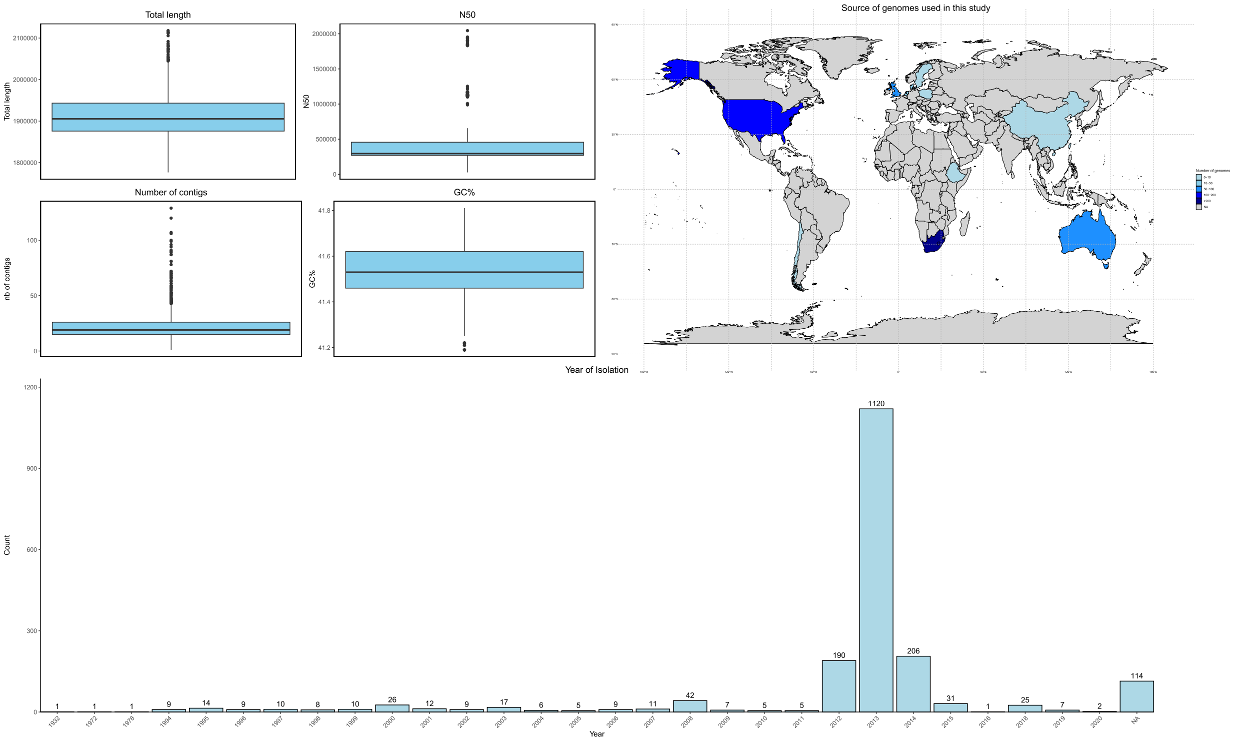

Supplementary Figure 1. Characteristics of the 1,913 *M. catarrhalis* genomes that passed quality control and were analysed in this study. Source data are provided as a Source Data file.

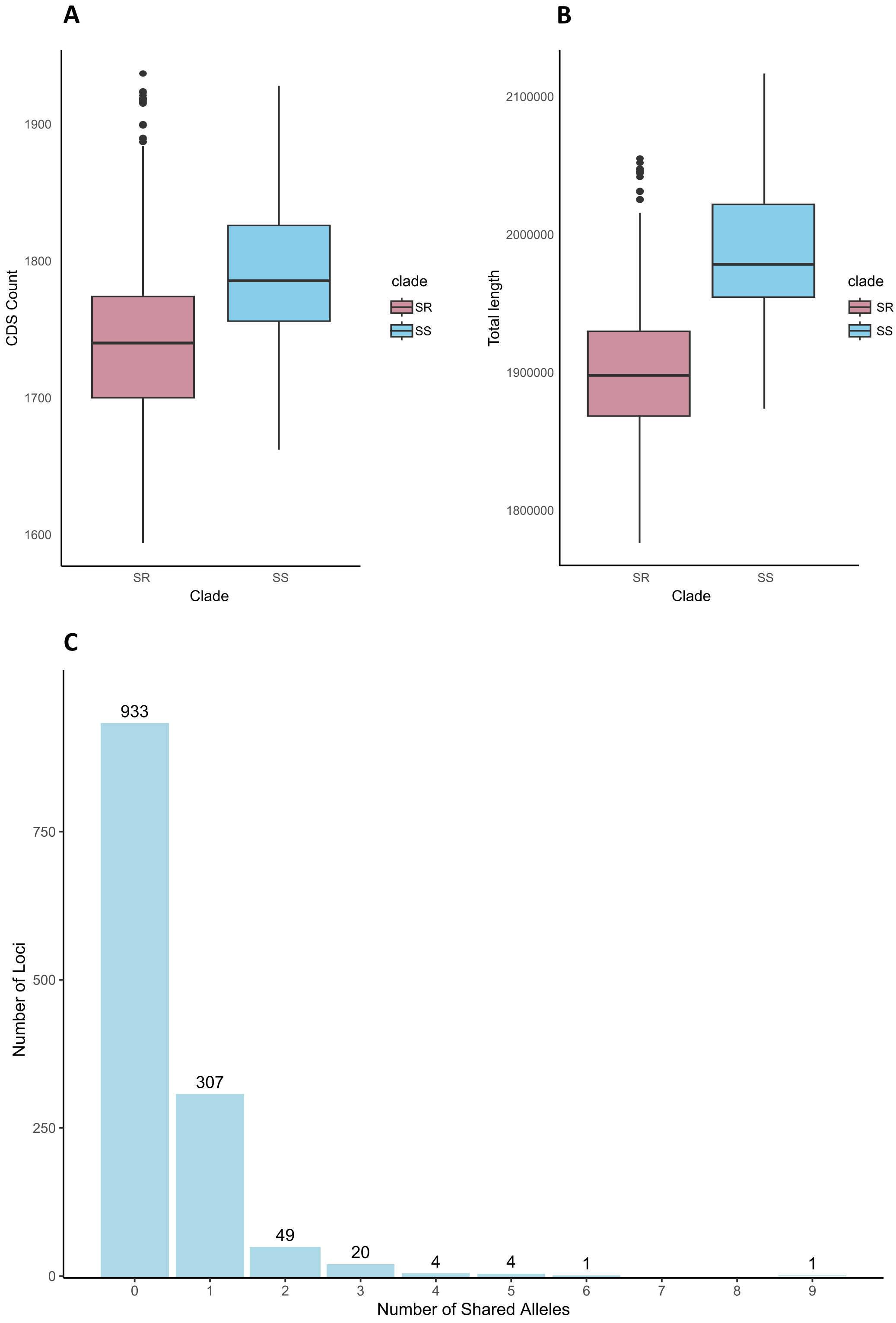

Supplementary Figure 2. (A) Box plot showing the distribution of coding sequences in SR and SS genomes. (B) Box plot comparing genome length distributions in SR and SS genomes. (C) Number of shared alleles between SR and SS among the 1,319 core genes. Note: CDS, coding sequence; SR, seroresistant; SS, serosensitive. Source data are provided as a Source Data file.

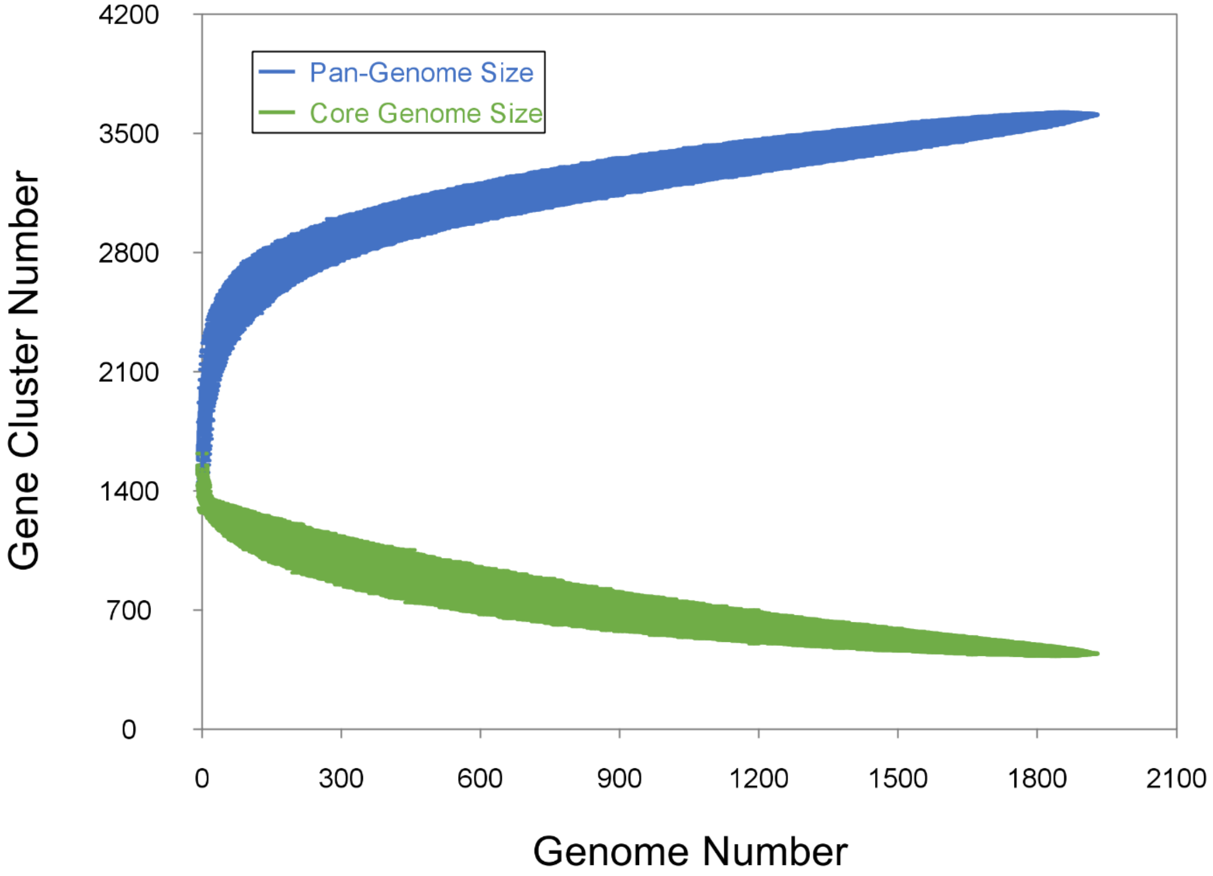

Supplementary Figure 3. Rarefaction curves of the pan-genome size and core genome size of *M. catarrhalis,* relative to the number of genomes analysed. Source data are provided as a Source Data file.

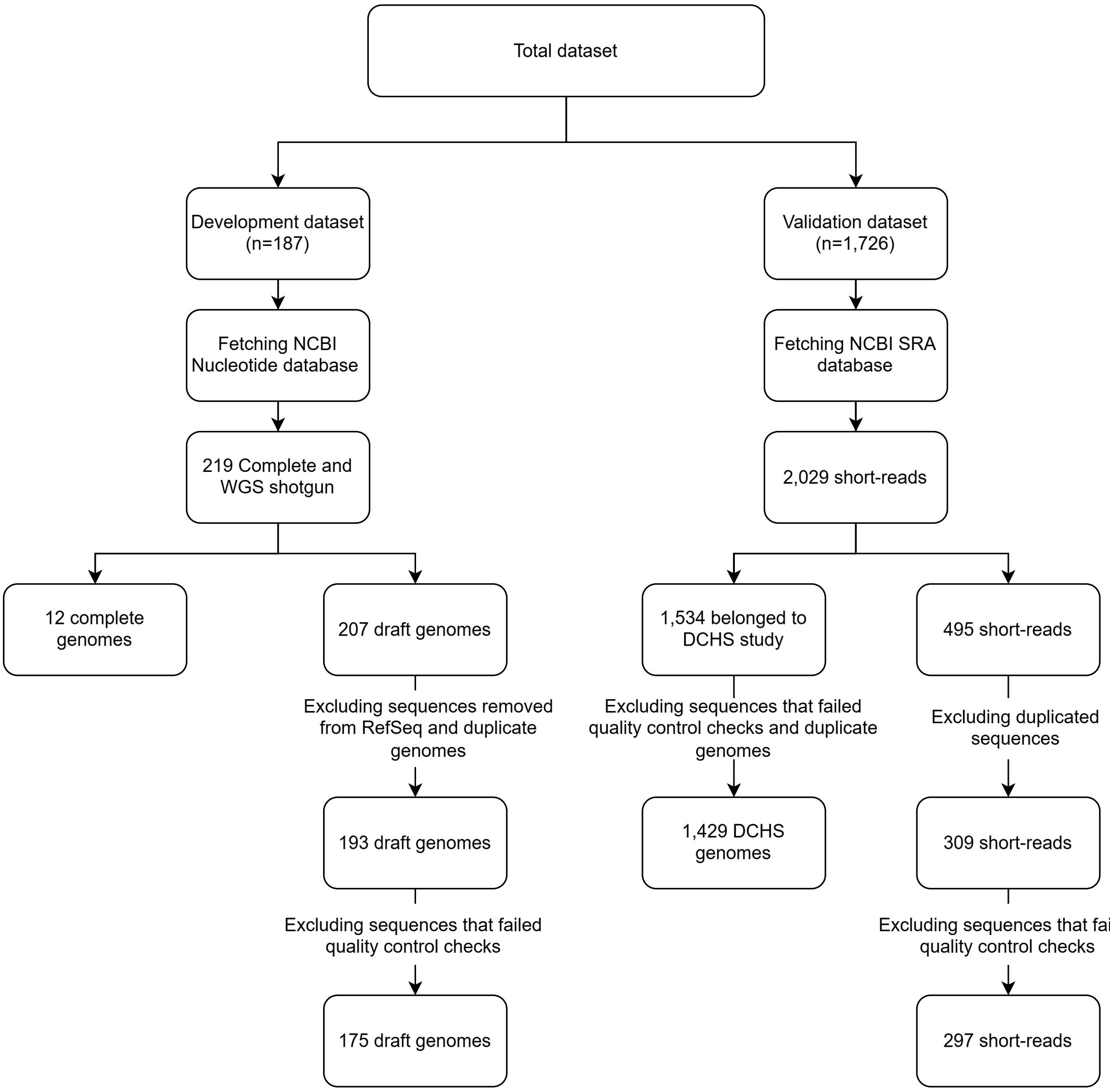

Supplementary Figure 4. Schematic overview of the selection and filtering process of *M. catarrhalis* genomes for the development and validation datasets. Source data are provided as a Source Data file.
