# Supplementary material for: Investigating the population structure of Moraxella catarrhalis using a cgMLST scheme and LIN code system
Source: Nat Commun. 2025 Oct 17;16:9137. doi: 10.1038/s41467-025-64487-8 (PMC12534458; doi:10.1038/s41467-025-64487-8)
Supplement: Supplementary file 2 — Description of Additional Supplementary Files [file 41467_2025_64487_MOESM2_ESM.pdf]

Title: Supplementary data 1
Description: Metadata and QUAST results for the final M. catarrhalis dataset used in this study.

Title: Supplementary data 2
Description: Clonal complex (CC) information from goeBURST.

Title: Supplementary data 3
Description: EggNOG mapper results (core genes).

Title: Supplementary data 4
Description: Per-genome assignment rates for all genes included in the core genome multilocus sequence typing scheme.

Title: Supplementary data 5
Description: 1,319 cgMLST profiles for M. catarrhalis genomes used in this study.

Title: Supplementary data 6
Description: Per-gene assignment rates for all genes included in the core genome multilocus sequence typing scheme.

Title: Supplementary data 7
Description: 1,319 cgMLST profiles for non-M. catarrhalis genomes used in this study.

Title: Supplementary data 8
Description: Pairwise distance matrix between non-M. catarrhalis and M. catarrhalis genomes used in this study.

Title: Supplementary data 9
Description: Genomic characteristic of M. catarrhalis genomes used in this study.

Title: Supplementary data 10
Description: Description of SR core genes.

Title: Supplementary data 11
Description: Description of SS core genes.

Title: Supplementary data 12
Description: Clade characteristics.

Title: Supplementary data 13
Description: Quality control thresholds used for the DCHS genomes.

Title: Supplementary data 14
Description: Details of genomes available in NCBI SRA database with information about excluded genomes.

Title: Supplementary data 15
Description: Details of genomes available in NCBI nucleotide/assembly database with information about excluded genomes.

Title: Supplementary data 16
Description: Assembly quality control thresholds used for the non-DCHS genomes.

Title: Supplementary data 17
Description: Details of Moraxella genomes available in rMLST database.

Title: Supplementary data 18
Description: Details of Moraxella genomes used to investigate the phylogenetic placement of M. catarrhalis among Moraxella species.

Title: Supplementary data 19
Description: Details of motifs, genes, primers used in this study.

Title: Supplementary data 20
Description: Accession numbers of genes sequences used in this study
