# Supplementary material for: Investigating the population structure of Moraxella catarrhalis using a cgMLST scheme and LIN code system
Source: Nat Commun. 2025 Oct 17;16:9137. doi: 10.1038/s41467-025-64487-8 (PMC12534458; doi:10.1038/s41467-025-64487-8)
Supplement: Supplementary file 23 — Reporting Summary [file 41467_2025_64487_MOESM23_ESM.pdf]

nature portfolio

Corresponding author(s): Angela B Brueggemann

Last updated by author(s): Jul 22, 2025

Reporting Summary

Nature Portfolio wishes to improve the reproducibility of the work that we publish. This form provides structure for consistency and transparency in reporting. For further information on Nature Portfolio policies, see our Editorial Policies and the Editorial Policy Checklist.

Statistics

For all statistical analyses, confirm that the following items are present in the figure legend, table legend, main text, or Methods section.

n/a	Confirmed	
☐	☒	The exact sample size (*n*) for each experimental group/condition, given as a discrete number and unit of measurement
☐	☒	A statement on whether measurements were taken from distinct samples or whether the same sample was measured repeatedly
☐	☒	The statistical test(s) used AND whether they are one- or two-sided<br>*Only common tests should be described solely by name; describe more complex techniques in the Methods section.*
☒	☐	A description of all covariates tested
☒	☐	A description of any assumptions or corrections, such as tests of normality and adjustment for multiple comparisons
☒	☐	A full description of the statistical parameters including central tendency (e.g. means) or other basic estimates (e.g. regression coefficient) AND variation (e.g. standard deviation) or associated estimates of uncertainty (e.g. confidence intervals)
☒	☐	For null hypothesis testing, the test statistic (e.g. *F*, *t*, *r*) with confidence intervals, effect sizes, degrees of freedom and *P* value noted<br>*Give P values as exact values whenever suitable.*
☒	☐	For Bayesian analysis, information on the choice of priors and Markov chain Monte Carlo settings
☐	☒	For hierarchical and complex designs, identification of the appropriate level for tests and full reporting of outcomes
☒	☐	Estimates of effect sizes (e.g. Cohen's *d*, Pearson's *r*), indicating how they were calculated

Our web collection on statistics for biologists contains articles on many of the points above.

Software and code

Policy information about availability of computer code

Data collection	We used sequenced and assembled M. catarrhalis genomes from an intensively sampled cohort of the Drakenstein Child Health Study. In addition, we included all M. catarrhalis genomes that were available in the public domain (NCBI nucleotide database and NCBI Squence Read Archive database) and passed quality control assessments.
Data analysis	VelvetOptimiser (v2.2.5), Velvet (v1.2), VelvetOptimiser (v2.2.6), SPAdes (v3.15.3), QUAST (v5.3.0), SSPACE (v2.1.1), GapFiller (v2.1.1), rMLST species tool implemented in PubMLST, chewBBACA software suite (v3.3.1), blastn (v2.13.0), PubMLST (BIGSdb automated curation tool, BIGSdb sequence tagging, cgMLST and LINcode assignment, in silico PCR), MAFFT (v7.525), Snp-sites (v2.5.1), IQ-TREE2 (v2.2.6), ClonalFrameML (v1.13), iTOL (v7), GrapeTree (v1.5.0), EggNOG-mapper (v2.1.12), ANIclustermap (v1.2), MSTclust (v0.21b), PHYLOVIZ (v2.00), R micropan (v.2.1) package, R mclust package (v6.1.1), PanGP (v1.0), ORF Finder (v1.8), MAST (MEME Suite v5.5.6), ResFinder (v4.5.0), R (v4.5.0), RStudio/2025.05.1+513, R tidyverse package (v2.0.0), R ggthemes package (v5.1.0), R scales package (v1.4.0), R patchwork package (v1.3.1), R dplyr package (v1.1.4), R ggplot2 package (v3.5.2), R readxl package (v1.4.5), R sf package (1.0.21), R openxlsx package (v4.2.8), R reshape2 package (v1.4.4). The code used to generate the figures is adapted from: https://github.com/brueggemann-lab/pgl_cgmlst_2024

For manuscripts utilizing custom algorithms or software that are central to the research but not yet described in published literature, software must be made available to editors and reviewers. We strongly encourage code deposition in a community repository (e.g. GitHub). See the Nature Portfolio guidelines for submitting code & software for further information.

Data

Policy information about availability of data

All manuscripts must include a data availability statement. This statement should provide the following information, where applicable:

- Accession codes, unique identifiers, or web links for publicly available datasets
- A description of any restrictions on data availability
- For clinical datasets or third party data, please ensure that the statement adheres to our policy

All genome sequences used in this study are publicly available in PubMLST (https://pubmlst.org/organisms/moraxella-spp) as well as the NCBI (SRA or nucleotide) databases. Short-read sequences of the DCHS samples have been deposited in the European Nucleotide Archive (ENA) under study number RJEB25371. Genome accession numbers are provided for each isolate in PubMLST and listed in Supplementary Data 1.

Research involving human participants, their data, or biological material

Policy information about studies with human participants or human data. See also policy information about sex, gender (identity/presentation), and sexual orientation and race, ethnicity and racism.

Reporting on sex and gender	Not applicable
Reporting on race, ethnicity, or other socially relevant groupings	Not applicable
Population characteristics	Not applicable
Recruitment	Not applicable
Ethics oversight	Not applicable

Note that full information on the approval of the study protocol must also be provided in the manuscript.

Field-specific reporting

Please select the one below that is the best fit for your research. If you are not sure, read the appropriate sections before making your selection.

[x] Life sciences [ ] Behavioural & social sciences [ ] Ecological, evolutionary & environmental sciences

For a reference copy of the document with all sections, see nature.com/documents/nr-reporting-summary-flat.pdf

Life sciences study design

All studies must disclose on these points even when the disclosure is negative.

Sample size	We included all M. catarrhalis genomes that were available in the public domain and passed quality control assessments in the development of this genotyping scheme, i.e. 187 out of 219 possible genomes from NCBI and 297 out of 495 possible genomes from the SRA that were not part of the DCHS, in addition to 1,429 out of 1,447 genomes of the DCHS.
Data exclusions	Samples were excluded from analyses if they were identified as the incorrect species, if they showed atypical features (inconclusive rMLST species identification result, unassigned rST or ST due to sequencing issues at one or more rST/ST loci), or if they had poor assembly statistics and/or features suggestive of a mixed culture or if they were duplicated (same sample name or biposample id).
Replication	All analyses related to several different genotyping techniques were repeated at least twice and we assessed concordance between the different classification outputs: CC, ST, rST, cgSTs and LINcodes. All attempts of replication were successful.
Randomization	This study involved analysis of publicly available genome sequences
Blinding	Blinding was not relevant as this study involved analysis of publicly available genomic datasets.

Reporting for specific materials, systems and methods

We require information from authors about some types of materials, experimental systems and methods used in many studies. Here, indicate whether each material, system or method listed is relevant to your study. If you are not sure if a list item applies to your research, read the appropriate section before selecting a response.

Materials & experimental systems

n/a	Involved in the study
☒	☐ Antibodies
☒	☐ Eukaryotic cell lines
☒	☐ Palaeontology and archaeology
☒	☐ Animals and other organisms
☒	☐ Clinical data
☒	☐ Dual use research of concern
☒	☐ Plants

Methods

n/a	Involved in the study
☒	☐ ChIP-seq
☒	☐ Flow cytometry
☒	☐ MRI-based neuroimaging

Plants

Seed stocks	Not applicable
Novel plant genotypes	Not applicable
Authentication	Not applicable
